# Effects of Physical Exercise on Walking Distance and Functional Limitations in Patients with Chronic Dyspnea

**DOI:** 10.3390/medicina61040636

**Published:** 2025-03-30

**Authors:** Kinga Vindis, Noemi Nemeth, Cristian Marge, Carmen Pantis, Mircea Gheorghe Pop, Manuela Simona Pop, Laura Ioana Bondar, Maria Carolina Jurcau, Katalin Babeș

**Affiliations:** 1 Doctoral School of Biomedical Sciences, Faculty of Medicine and Pharmacy, University of Oradea, 410073 Oradea, Romania; ujj.kinga@student.uoradea.ro (K.V.); nemeth.noemi@didactic.uoradea.ro (N.N.); bondar.lauraioana@student.uoradea.ro (L.I.B.); 2 Department of Psycho Neuroscience and Recovery, Faculty of Medicine and Pharmacy, University of Oradea, 410073 Oradea, Romania; marge.cristian@uoradea.ro; 3 Department of Surgical Disciplines, Faculty of Medicine and Pharmacy, University of Oradea, 410073 Oradea, Romania; gmpop@uoradea.ro; 4 Department of Biology and Life Sciences, Faculty of Medicine, Vasile Goldiș Western University of Arad, 310048 Arad, Romania; 5 Faculty of Medicine and Pharmacy, University of Oradea, 410073 Oradea, Romania; 6 Cardiology Department, Faculty of Medicine and Pharmacy, University of Oradea, 410073 Oradea, Romania; katalin.babes@didactic.uoradea.ro

**Keywords:** chronic dyspnea, physical workout, functional limitation, walking distance

## Abstract

*Background and Objectives*: Chronic dyspnea is a common clinical manifestation in patients suffering from cardiovascular and respiratory diseases globally, representing an independent predictor of mortality for these patients. In addition, it may be a symptom associated with other conditions such as anemia, physical deconditioning, or anxiety. *Methods*: A prospective study was conducted, between 1 January 2021 and 30 June 2022, at the Medical Recovery Section from “Dr. Pop Mircea Municipal Hospital Pop Mircea” in Marghita. A total of 163 consecutive patients with chronic dyspnea of various etiologies were evaluated for inclusion in the study. Patients who met the inclusion criteria followed a personalized physical training program of variable duration (between 20 and 40 min) up to the limit of exercise tolerance (grade 3–4 modified Borg scale or up to 70% of maximum heart rate, calculated with the formula 220 age in years); the first 10 days, the training was supervised by a physiotherapist, then patients followed a program of 30 min of exercise 5 days/week at home for 3 months. Assessments, performed at inclusion and after 3 months of training, consisted of the 6 min walk test (6MWT) and the London Chest Activity of Daily Living (LCADL) scale. *Results*: Pulmonary etiology is the most common cause of dyspnea in the cohort (61.65%). The number of patients without ventilatory defects is 56, or 38.35%. The mean value of initial functional limitation (LCADL1) improved significantly after 3 months (LACDL2) of rehabilitation treatment (38% versus 26.5%); at the same time, the mean walking distance (6MWT) increased by 76 m. *Conclusions*: An adequate rehabilitation program and sedentary lifestyle change significantly reduce the functional limitation of the patient with chronic dyspnea and increase walking distance. Predictors for 6MWT gait test are age, LCADL score, dyspnea level, and cardiac etiology of chronic dyspnea.

## 1. Introduction

Dyspnea is a clinical manifestation frequently associated with chronic diseases, with a prevalence of 10% in the general population [1]. According to some authors, the incidence would be approximately 25% of the patients seen in consultation at the outpatient clinic [2]. Reflecting a deterioration in cardiorespiratory function, it has a negative impact on tolerance to physical activities, and on the ability to perform professional tasks, ultimately affecting quality of life, both personally and professionally. There is an international concern to ensure the functional autonomy of patients with chronic dyspnea, which could considerably reduce social, economic, and individual costs.

The etiology of chronic dyspnea is generally multifactorial, with a high prevalence of pulmonary or cardiac causes [3]. Along with bronchial asthma, heart failure, ischemic heart disease, chronic obstructive pulmonary disease (COPD), pneumonia, and psychogenic conditions, which account for 85–90% of the cases of dyspnea, there are other causes, such as anemia, obesity, physical deconditioning [4], gastric, neuromuscular disorders, and uncommon conditions [5]. The etiology of dyspnea has been shown to have several causes in over one-third of patients. The diagnosis is clinical in over 60% of cases. Additional paraclinical investigations are required to support the diagnosis [5].

Dyspnea is a complex symptom that indicates a potential threat to homeostasis, occurring due to impairment of the cardiovascular and respiratory systems, but also due to metabolic disturbances, neuromuscular disorders, or psychogenic disorders, caused by a mismatch between pulmonary ventilation and the desire to breathe. The dissociation between these two aspects is the result of a mismatch between receptors in the airways, lungs, and chest wall and central respiratory motor activity. Dyspnea is influenced by afferent signals (information about blood gas levels and lung status), efferent signals (commands to respiratory muscles, diaphragm), and central brain processing, which compares these signals. When there is a mismatch between the need for ventilation and the ability to breathe, the intensity of dyspnea increases, and the sensory cortex activates the conscious sensation of exertion and shortness of breath. The psychological component also plays a role, as some people may be aware of their breathing without feeling discomfort [6]. At the same time as physiopathological changes occur in the lungs, there are also changes in the cardiovascular system. One can notice an increase in right ventricular afterload, followed by right ventricular hypertrophy, and finally, right ventricular failure [7], which will affect the mass, geometry [8], and by implication, left ventricular systolic function [9]. These changes will cause the left heart to be overloaded and exceed its exercise capacity.

Emotion influences the neural processing of respiratory sensations and interacts with non-emotional mental processes such as attention and expectation, affecting aversive experiences such as pain and dyspnea. Attentional resources are allocated according to emotional salience, and focusing on the threat of dyspnea may reduce performance in daily tasks. High dyspnea-related anxiety impairs the ability to allocate attention to cognitive tasks, especially in negative emotional contexts. Although exercise may benefit cognitive function, its impact on dyspnea patients needs to be studied. Attention allocation is crucial in treatment and patients with pulmonary disease are encouraged to focus on breathing. Inter-interoceptive self-awareness may reduce the intensity of dyspnea, but negative emotions may hinder this cognitive manipulation [10].

The literature specifically emphasizes the benefits of exercise training in the management of pulmonary dyspnea, especially in patients with COPD. Exercise limitation is closely related to disability and is a significant indicator of poor health as well as a risk factor for premature mortality. Factors influencing exercise restriction vary from patient to patient, including respiratory pathologies, associated cardiovascular and neurological conditions, and musculoskeletal dysfunction. The implementation of a rehabilitation program with a multidisciplinary approach for 6 to 8 weeks demonstrated a significant improvement in dyspnea and exercise tolerance. There was also an improvement in mental well-being and a reduction in hospitalizations. However, despite continued support for pulmonary rehabilitation in the literature, debates remain about the specific content of pulmonary rehabilitation and its benefits for the specific patient [11]. The American Thoracic Society/European Respiratory Society suggests the use of pulmonary rehabilitation (PR) for all patients who require this intervention, as it can bring significant benefits in terms of quality of life. PR applied to patients with respiratory pathology will reduce dyspnea and increase exercise performance, implicitly improving the quality of life. The highest addressability is for patients with COPD. PR programs include assessing the patient’s condition, and implementing physical training, along with nutritional interventions and psychosocial support [12].

Regardless of the respiratory function, muscle fatigue of the muscles in the lower limbs is frequently associated with patients with cardiorespiratory pathology. In terms of the skeletal muscle fiber, a series of transformations occur, determined by the deconditioning of these patients. An important witness is the strength of the quadriceps, which correlates with the exercise capacity [11].

The traditional program, used for patients with chronic dyspnea, consists of aerobic exercises and resistance training for the upper and lower limbs, which is associated with walking and cycle ergometer training, instituted progressively to increase muscle conditioning and cardiorespiratory fitness [11]. This endurance training applied for 3 months reveals proven benefits on exercise capacity, increasing the strength of the quadriceps muscle (known dysfunction in COPD) [13,14].

Patients with chronic dyspnea, regardless of etiology, are forced to reduce their daily activities due to chronic shortness of breath [15], thus creating a vicious circle that promotes physical deconditioning and social isolation [16]. The London Chest Activity Scale of Daily Living (LCADL) is a questionnaire that measures functional limitation due to dyspnea experienced during activities of daily living, such as personal care, housework, physical exercise, and recreation [17]. The 6 min walk test (6MWT) is recommended as an effective tool to assess treatment efficacy by examining pulmonary function and exercise capacity [18]. Because the test incorporates a level of physical activity comparable to that of daily tasks, it can accurately indicate patients’ pulmonary function and exercise capacities during daily routines [18].

Although exercise has been shown to have a positive impact on cognitive function, there is a need to investigate whether this effect also applies to patients suffering from dyspnea [10]. Based on the hypothesis that a progressive physical training program, adapted to cardiopulmonary reserve, reduces functional limitation in patients with chronic dyspnea, this study aims to (1) evaluate and compare the benefit of traditional physical training in patients with chronic dyspnea of different etiologies, by assessing walking distance; (2) compare the evolution of dyspnea grade in ADLs after physical training; (3) identify predictive factors associated with the 6MWT gait test in patients with chronic dyspnea. Also, an original aspect will be followed, namely the effects on each given ADL domain (personal care, domestic chores, exercise, and leisure activities).

## 2. Materials and Methods

### 2.1. Study Design

A prospective study was carried out, between 1 January 2021 and 30 June 2022, within the Medical Recovery Department at “Dr. Pop Mircea” Municipal Hospital in Marghita. A total of 163 consecutive patients with chronic dyspnea of various etiologies were evaluated for enrollment in the study.

#### Inclusion/Exclusion Criteria

A total of 163 patients with chronic dyspnea of cardiovascular, pulmonary, or other etiology were selected. The etiologic stratification was performed based on anamnesis, clinical and paraclinical examination (spirometry). Spirometry was conducted using a Contec SP10 Spirometer, following the guidelines set by ATS/ERS. The patients were previously evaluated from the point of view of the underlying pathology. They continued the chronically administered medication without any intervention (except in cases of decompensation).

**Figure 1:**
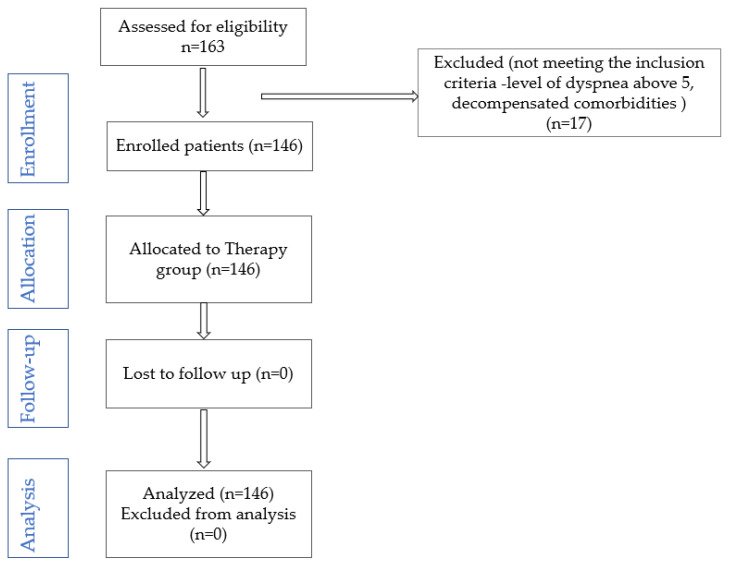
CONSORT flow diagram of the study.

Exclusion criteria: age under 18 years, psychogenic dyspnea, decompensated comorbidities that prevent the establishment of the rehabilitation program, dyspnea above level 5 (modified Borg scale). After applying the exclusion criteria, 146 patients remained in the study (Figure 1). All patients were included in a progressive physical training program, in terms of intensity and duration (cycling, progressive resistance exercises), depending on individual tolerance, as follows: 10 days in the physical therapy room, followed by the recommendation to continue training to increase exercise tolerance, at home, three times a week (walking, resistance exercises established together with the rehabilitation physician, depending on individual tolerance).

### 2.2. Study Tools

The London Chest Activity of Daily Living (LCADL) scale is an instrument designed to measure the degree of dyspnea experienced during daily activities. It covers four domains: self-care, household activities, exercise, and recreation. Considered to be an affordable and simple-to-use tool, the LCADL may be a viable clinical option for assessing and monitoring the functional impact of dyspnea. The LCADL scale is a self-report, valid and reliable for dyspnea assessment during ADL [19], which measures the functional limitation caused by dyspnea experienced [20] during daily living activities (personal care, domestic chores, exercise, and leisure activities). The LCADL includes 15 items, quantified with the Likert scale. The final score (0–75) is obtained by adding up the scores on each domain. The higher the value, the more pronounced the functional limitation. For greater accuracy in interpretation, we will use the values expressed in percentages [21].

The modified Borg dyspnea scale is a self-reported tool used to assess the level of breathlessness during physical activity. While it is a subjective measure of dyspnea intensity, the scale has proven reliable in quantifying breathlessness in patients participating in clinical trials, particularly those who undergo a six-minute treadmill test. To assess the patient’s perceived effort through dyspnea, the modified Borg scale was used (protocol 22 April 2021); thus, the value 0 means no dyspnea, 1—Vaguely noticeable, 2—Very light, 3—Mild to moderate, 4—Moderate, 5–6—Moderate-severe, 7—Severe, 8—Very severe, 9–10—Maximal shortness of breath [22].

The 6 min walk test (6MWT), introduced in 2002 for the evaluation of patients with cardiopulmonary pathologies, is a submaximal exercise test [23] in which patients determine the intensity of their effort. They walk a flat distance, at a self-imposed pace, for 6 min. This submaximal test quantifies the walked distance [24]. The test may be interrupted if dyspnea, fatigue, increased heart rate, and decreased O_2_ saturation occur <85% [25].

The patients included in this study were evaluated at the beginning of the study and 3 months after the institution of the training program. The assessments consisted of clinical evaluation, a 6MWT test, an LCADL questionnaire, and a questionnaire on employment status data.

All patients performed continuous physical training, with progressive duration from 20 min to 40 min, up to the limit of tolerance (fatigue, dyspnea grade 3–4) or up to a frequency of 70% of maximum heart rate (220—age in years). The training sessions were performed in the physiotherapy room, 5 days/week, for 2 weeks, with the following structure: warm-up period (cycle ergometer, progressive from 5 min to 10 min, 25 watts), the actual training (25 min, active mobilizations, progressive resistance exercises, starting from 0.5 kg for limb muscle toning, resistance exercises for inspiratory and expiratory muscle toning, including exercises with weights on the abdomen for diaphragm toning) and a 5 min cooling-down period. At discharge, patients were advised to continue training 5 times a week, 30 min daily, for 3 months, respecting the same effort limits applied in the physiotherapy room. To increase adherence to the exercise program, patients were given a training plan and were encouraged to keep a diary or purchase a fitness bracelet (which has the property to monitor a number of parameters: heart rate during exercise, number of steps, etc.).

### 2.3. Ethical Approval

This study was approved by the Ethics Commission of County Hospital (Approval No. 4842/3 December 2020) and complies with the Declaration of Helsinki of the World Medical Association.

### 2.4. Sample Size

To determine the sample size, we considered the following variables: *p*—the probability of occurrence of the phenomenon, where 0 < *p* < 1; q—the counter-probability, calculated as q = 1 − *p*; t—the probability factor; x—the margin of error; N—the total population size. We used the following formula to determine the sample size (*n*): *n* = t2 pq/(x2 + t2 pq/N). Using the above calculations, we determined that the sample size (*n*) required for our study was 85.

### 2.5. Statistical Analysis

JASP version 0.18.1.0 [26] was used for statistical processing of the data. Means and standard deviations were determined and the Student *t*-test and Wilcoxon Signed Ranks Test were run. A multiple linear regression model was fitted to identify factors influencing 6MWT. One-way analysis of variance (ANOVA) allowed for comparison results according to etiology (pulmonary, cardiac). Repeated-measures ANOVA analysis was used to identify differences in measurements over time.

The statistical significance was set for *p* < 0.05.

## 3. Results

### 3.1. General Characteristics

The general characteristics of the cohort evaluated are listed in Table 1. The mean age of the patients included in this study was 58.19 ± 7.58 years. The data presented in the tables suggest a predominance of male patients (63.70%), with a predominantly urban environment (75.35%). Over 50% of the evaluated patients are active people, presenting an employee profile (56.85%). The increased body mass index is found in about 60% of the subjects included in the study. Regarding the possibility of performing ADLs, Table 1 suggests that 8.90% of the recruited patients are dependent.

### 3.2. Distribution According to Etiology and the Presence of the Ventilatory Defect

Dyspnea of pulmonary cause is the most frequent cause of dyspnea at the cohort level (61.65%). The number of patients without ventilatory defects is 56, i.e., 38.35%. The distribution of patients according to dyspnea assessed at rest (modified Borg scale) is included in Table 2. More than 60% of the patients included in the study present level 1 and level 2 dyspnea.

### 3.3. Evaluation of Functional Limitation Determined by Dyspnea Felt with the Help of LCADL Test and Walking Distance—6MWT

The analysis of the data in Table 3 indicates a low LCADL1 score, obtained at the first determination (28.53 ± 13.26), which suggests a functional limitation caused by dyspnea during daily activities. The average percentage value was 38% of the maximum value. At the second determination, the LCADL2 score improves significantly (*p* < 0.001), with functional limitation representing 26.5%. The average distance at the second evaluation, performed 3 months later, is approximately 76 m above the average of the first determination

**Figure 2:**
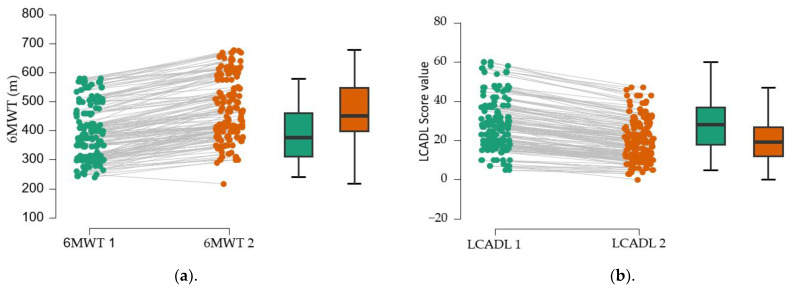
Evolution of the parameters assessed between the 2 determinations: (**a**). 6 min walk test; (**b**). LCADL score. m—meter; 6MWT 1—6 min walk test, first evaluation; 6MWT 2—6 min walk test, second evaluation; LCADL 1—London Chest Activity of Daily Living, first evaluation; LCADL 2—London Chest Activity of Daily Living, second evaluation; black vertical lines—confidence intervals (95%).

The normality test indicates a normal distribution (*p* > 0.05). To identify the differences in the measurements of the 6MWT test between the two determinations, we used the *t*-test (Figure 2), which suggests a significant difference between the means of the two determinations (*p* < 0.001).

**Figure 3:**
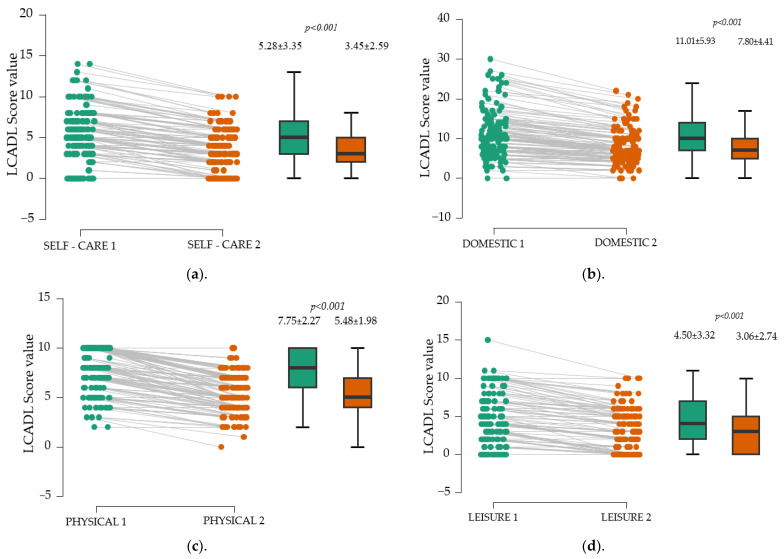
Evolution of LCADL score by domains. (**a**). Personal care. (**b**). Domestic activities. (**c**). Performing physical training. (**d**). Performing leisure activities. London Chest Activity of Daily Living domain scores: SELF-CARE 1—first evaluation; SELF-CARE 2—second evaluation; DOMESTIC 1—first evaluation; DOMESTIC 2—second evaluation; PHYSICAL 1—first evaluation; PHYSICAL 2—second evaluation; LEISURE 1—first evaluation; LEISURE 2—second evaluation; black vertical lines—confidence intervals (95%).

The use of the paired *t*-test allowed to highlight the benefits of physical training in patients with dyspnea. The analysis of the data in Figure 3 suggests significant differences in each domain (*p* <0.001). The average values in each area decrease, which means that personal care, the performance of domestic activities, the possibility of exercising, and leisure activities increase.

### 3.4. Evolution of the 6MWT Test According to the Dyspnea Etiology

To assess significant differences between the initial and final determinations of the 6MWT test, a repeated-measures ANOVA was performed. The data analysis in Table 4 indicate significant differences between the two determinations (*p* < 0.001).

Post hoc analysis (Table 5) suggests a mean difference of 76.582, which indicates the existence of a considerable difference between the two levels compared (p_bonf_ < 0.001). The high t-value of 24.795 further supports the significance of the difference, indicating a strong effect size.

**Figure 4:**
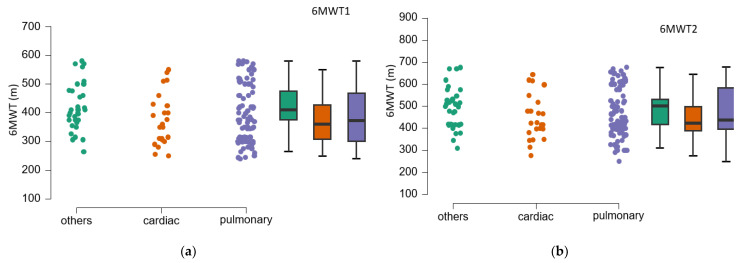
Mean values of the 6MWT test according to etiology. (**a**). Initially; (**b**). After 3 months. 6MWT1—6 min walk test, first evaluation; 6MWT2—6 min walk test, second evaluation; black vertical lines—confidence intervals (95%).

One-way analysis of variance (ANOVA) allowed for comparison results according to etiology. The mean walking distances and their standard deviations from the initial and final 6MWT test, categorized by etiology, are presented in Table 6 and Figure 4.

**Figure 5:**
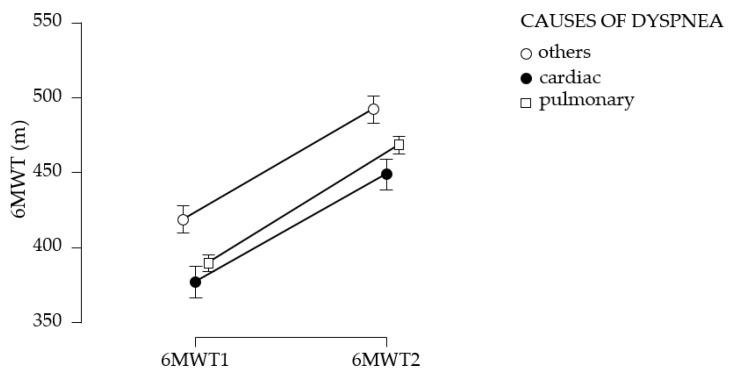
6MWT test results differentiated by etiology. black vertical lines—confidence intervals (95%).

Figure 5 suggests the comparative initial and final walking distance as a function of evolution.

Post hoc testing (Table 7) shows that there is no significant difference between the two determinations according to etiology (*p* > 0.05).

### 3.5. LCADL Correlated with 6MWT

The analysis of correlation data (Table 8) shows that the LCADL1 score (initial) correlates strongly negatively with walking distance (r = −0.794) and moderately positively with age (r = 0.484). A moderate and negative correlation was also identified between the walk test and age (r = −0.615). At the same time, data analysis suggests a low–moderate positive correlation between LCADL1 and age (r = 0.484). The association between the LCADL score and walking distance was also maintained at the 3-month follow-up (r = −0.778).

**Figure 6:**
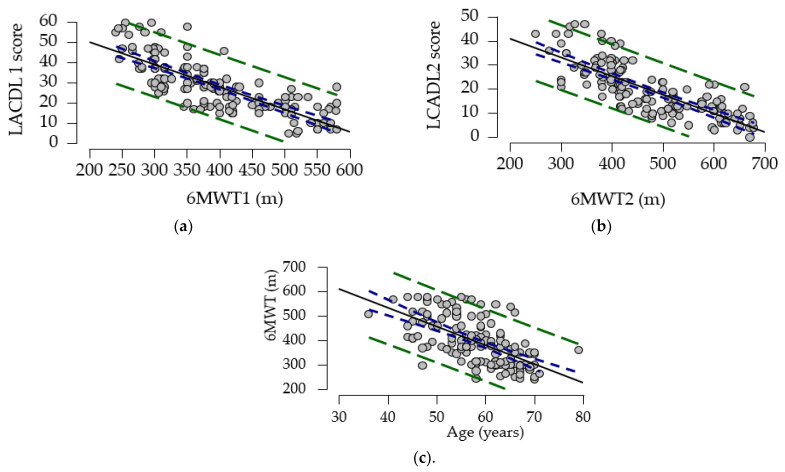
(**a**). 6MWT−LACDL1 score correlation; (**b**). 6MWT–LACDL2 score correlation. (**c**). 6MWT–Age correlation. m—meter; 6MWT—6 min walk test; LCADL1—London Chest Activity of Daily Living, first evaluation; LCADL2—London Chest Activity of Daily Living, second evaluation; blue dotted lines—confidence intervals (95%); green dotted lines—prediction intervals (95%).

The significant effects are depicted in Figure 6.

### 3.6. Identification of Predictive Factors for the Walk Test

The multivariate regression model explains 75.1% of the total variability of the walk test and is statistically significant [F(12, 133) = 33.404, *p* < 0.001]. Next, we inspected the model’s coefficients to see which of the predictors contributed significantly to the explained variable (Table 9). The significant predictors identified are age, LCADL score, dyspnea score, and cardiac etiology.

**Figure 7:**
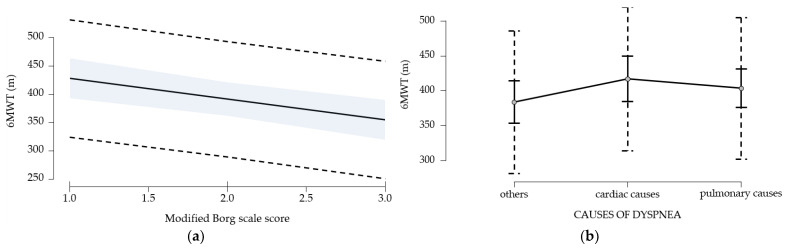
(**a**). Regression diagram 6MWT—modified Borg scale. (**b**). Regression diagram 6MWT—chronic dyspnea etiology. (**a**). blue line—confidence intervals (95%); dotted line—prediction intervals (95%); (**b**). dotted line—prediction intervals (95%); black lines—confidence intervals (95%).

The significant effects are depicted in Figure 7.

## 4. Discussion

This study aims to fill gaps in the literature on the benefits of physical training in patients with chronic dyspnea. Based on the hypothesis that a progressive physical training program, adapted to cardiopulmonary reserve, reduces functional limitation in patients with chronic dyspnea, the effects of a traditional physical training program on walking distance and daily activities, both globally and by activity domains, were analyzed. The originality of this study lies in the attempt to stratify these benefits according to the etiology of chronic dyspnea. Analysis of the results supports the hypothesis formulated; walking distance traveled significantly increased, regardless of the etiology of dyspnea. In addition, the LCADL score improved at the cohort level, both overall and in all four domains assessed.

The results are supported by several published studies, which show that the increase in walking distance and the reduction in dyspnea is achieved following appropriate physical training applied to patients with chronic dyspnea [27,28], but most studies refer to respiratory pathology (COPD). The physical training used to increase the exercise capacity of patients with chronic dyspnea is diverse, from traditional exercises to Tai Chi, Yoga, Daoyin, and even walking [14]. In this study, the physical training followed was traditional. Traditional endurance training consists of long sessions of moderate-intensity exercise, such as walking, cycling, or jogging, performed at a consistent pace for an extended duration, typically 30 min or more. This type of training is effective in enhancing aerobic capacity and overall fitness, particularly for individuals with chronic diseases. Long-term exercise leads to a more efficient delivery of oxygen to the muscles and enhances endurance capacity. Additionally, training with heavy weights promotes muscle growth and toning. However, recent studies in high-intensity training with short intervals and low-load exercises—aimed not at increasing muscle size—challenge the traditional view of training specificity. To gain a better understanding of the muscle adaptations resulting from different types of exercise, it is essential to explore the molecular mechanisms that influence changes in muscle phenotype [29]. High-intensity interval training (HIIT), on the other hand, involves short bursts of intense exercise followed by periods of rest or low-intensity activity. Research on the relationship between chronic obstructive pulmonary disease (COPD) and the effects of HIIT in pulmonary rehabilitation is still limited. However, one study has demonstrated that COPD patients can improve their functional capacity after 12 weeks of high-intensity training [30]. Another study also reported positive changes in systolic function and cardiovascular values following HIIT and moderate exercise. A systematic review found that patients with moderate to severe COPD can perform HIIT, leading to improvements in ventilatory parameters and a reduction in exercise-associated dyspnea [31]. HIIT leads to positive changes in ventilatory parameters and a reduction in exertional dyspnea [32]. The findings of the systematic review published by J. R. Adolfo (2019) suggest that the effects of HIIT are similar to those obtained with continuous exercise in terms of relative VO2max, absolute VO2max, and cardiovascular variables among patients with COPD. Regarding the type and intensity of physical activities recommended in pulmonary rehabilitation programs, research has shown favorable results for both HIIT and continuous moderate-intensity exercise in COPD patients [31]. The narrative review, published by M Emtner and K Wadell (2016), showed that aerobic and resistance training lead to increased quality of life in patients, as well as decreased dyspnea, anxiety, and depression [33], results supported by the data obtained in this study.

The analysis of the data suggests that, although according to the average age, the patients included in the study are middle-aged, only about 50% of them have the status of an employee, meaning that chronic dyspnea, regardless of the etiology, significantly limits their ability to carry out a professional activity. Domestic activities can be performed by more than 85% of the recruited patients, but only about 10% of patients are able to practice hobbies prior to the onset of the disease. Pulmonary etiology is present in over 60% of cases (COPD, asthma), and cardiac pathology in about 30% (heart failure). A study conducted on 120 patients with COPD [34], published by H. Carette (2019), suggests that 53% of the patients treated with inhaled medication have severe chronic dyspnea, and 40% have undergone a pulmonary rehabilitation program. In our study, the incidence of dyspnea level 3 is 37.67%.

Obstructive ventilatory defect is present in 35.61% of recruited cases. Restrictive ventilatory defect, present in 38.35% of cases, was determined by the presence of kyphoscoliosis and associated neurological diseases. In about 8% of cases, chronic pulmonary pathology was associated with kyphoscoliosis or neurological diseases.

The meta-analysis published by Ahmed I. et al. (2022), which monitored the effect of PR on dyspnea, fatigue, and exercise capacity, identified five clinical trials (N = 449 patients, in patients with acute and/or chronic COVID-19) that suggested significant benefits of PR on dyspnea, fatigue, and exercise capacity [25]. The PR program is structured, similar to the program used in our study, in the three specific stages of a training program (warm-up phase—consisting of active mobilizations, the actual training phase—consisting of strength and gait training; at the end, stretching exercises are performed to allow blood pressure and heart rate to return to normal); the difference is the recommendation to be used 4 times/week compared to our study, in which the recommendation is 5 times/week. In both cases, the benefit of applied physical training was demonstrated.

The assessment of functional limitation, as determined by dyspnea experienced during daily activities with the LCADL test, suggests a 38% limitation at first determination. Following the establishment of a pulmonary rehabilitation program, followed intensively for 10 days, then 3 times a week for 3 months, an 11.5% improvement in functional capacity was observed. This improvement is measured by the 6MWT gait test; walking distance increases by 76.58 m at 3 months after the training. The study suggests the improvement of dyspnea, regardless of the cause; the best effects, in terms of increased walking distance, were obtained in patients with dyspnea of etiology other than cardiac or pulmonary, followed by patients with pulmonary involvement; patients with cardiac involvement have the poorest results. A study published by C. B. Moreno et al. (2018) suggested increased physiological trainability in patients with COPD (N = 193), as evidenced by the 6MWT gait test; exercise tolerance improved and walking distance increased by 20.76 m [28]. In our study, the walking distance increased significantly (76.58 m) compared to the results of the study published by C. B. Moreno et al. because 55 of the patients in the study only underwent an educational program of disease awareness, healthy habit discussions (nutrition, physical training, physical activity in daily life), exact-herbs awareness, and vaccination. Another explanation would be that our study did not exclude patients with severe dyspnea, above level 5 on the modified Borg scale, and the group of patients was inhomogeneous in terms of dyspnea etiology.

The study conducted on 18 patients with COPD published by N Miyahara et al. (2000) supports the benefits of physical training (gymnastics to tone the respiratory muscles, the skeletal muscles, and ergometer cycling) on dyspnea, fatigue, and emotional condition for 3 weeks [35]. Another study conducted on 30 patients (15 patients—control group), published by M. S. Ali (2014), suggests the benefits of physical training (20 min of walking, ergometer cycling, and resistance exercises, three sessions/week, three consecutive weeks) on 6MWT and exercise capacity [36].

Patients experiencing dyspnea of cardiac origin, particularly those with heart failure (HF), often face limitations in their exercise capacity. Regarding the improvement of dyspnea and exercise capacity in patients with HF, published studies highlight the critical role of exercise [37,38,39]. For instance, a study by Donna M. Mancini et al. (1995) involving 14 HF patients undergoing specific respiratory muscle training for 3 months (90 min, three times a week) indicated an increase in exercise capacity as measured by the 6-minute walk test (6MWT) [38]. Similar findings were reported by B. Pozehl et al. (2008) [40], where 15 HF patients participated in aerobic and endurance training three times a week for six months, demonstrating notable improvements in dyspnea and fatigue compared to a control group without rehabilitation. Furthermore, the study by Alexis L. Beatty et al. (2012, n = 556) [41] found that the distance achieved in the 6MWT can predict subsequent cardiovascular events. The repeated-measures ANOVA method, applied to the data collected in our study, suggests the benefits of physical training on chronic dyspnea, highlighting the differences obtained at 3-month vs. first assessment. Moreover, statistical analysis of the data suggests that the mean walking distance obtained in the initial and final 6MWT test did not differ according to the etiology of dyspnea (*p* > 0.05).

Besides enhancing walking distance, a secondary objective of our study was to evaluate the LACDL score across different domains. After three months, significant improvements were observed in all four assessed areas of the LACDL score—personal care, domestic chores, exercise, and leisure activities (*p* < 0.001).

To explore the relationship between LCADL 1, 6MWT, and age, we calculated the correlation coefficient. Our study showed a strong negative association between the gait test and LCADL score compared to the results of a cross-sectional study (N = 44) by Ozsoy I. et al. (2019), who reported a low to moderate association [42]. To further elucidate the relationships between these variables, a multivariate regression model was employed. This model identified significant predictors for the 6MWT, including age, LCADL score, dyspnea score, and cardiac etiology. Similar associations between the 6MWT and LCADL were noted in the research conducted by Ozsoy I. et al. [42]. Moreover, in a study by Y. Al Chikhanie et al. (2021), age and dyspnea levels were also identified as predictors for the 6MWT in patients with COPD [43]. These findings collectively emphasize the importance of targeted exercise interventions in improving outcomes for patients with heart failure and related conditions.

### Strengths and Limitations of the Study

This study is among the few studies that assess functional limitation by domains—personal care, domestic chores, exercise, and leisure activities. It is also the first study in Romania that aims to identify the predictive factors that limit the 6 min walk test. The main limitations of the study are primarily due to the inhomogeneity of the study group, from an etiological point of view, the lack of determination of the grip strength of the hands and the strength of the quadriceps muscle. Monitoring adherence to physical training at home is a limitation of the study, but to reduce this, patients were encouraged to keep a diary or purchase a fitness bracelet. The exclusion of patients with high effort scores (Borg score > 5) may introduce a selection bias, causing the results to be influenced by the characteristics of the patients included in the study. Thus, an improvement in outcomes may be observed that is not representative of the entire patient population. The study findings may apply only to a small subset of patients.

## 5. Conclusions

Chronic dyspnea causes functional limitation, expressed by reduced ability to perform activities of daily living (personal care, housework, physical exercise, and leisure activities). A proper rehabilitation program and sedentary lifestyle change significantly increase walking distance and reduce functional limitation, regardless of the etiology of chronic dyspnea. Predictors for the 6MWT gait test are age, LCADL score, dyspnea level, and cardiac etiology of chronic dyspnea. The analysis of the impact of chronic dyspnea on functional capacity is essential for the development of effective symptom control strategies, to increase the independence of patients with chronic dyspnea. This study supports the benefits of traditional physical training applied to patients with chronic dyspnea of different etiologies, on the four domains of daily activities (personal care, domestic chores, exercise, and leisure activities). Future studies should include a control group to validate the results and provide a clearer understanding of causal relationships.

## Figures and Tables

**Table 1:** Baseline characteristics.

Parameters	Study Group
**DD**	
Age, years, mean ± SD	58.19 ± 7.58
Urban environment, N (%)	110 (75.35)
Female gender, N (%)	53 (36.30)
Employment profile, N (%)	83 (56.85)
**BMI**	
Healthy weight, N(%)	56 (38.35)
Underweight, N (%)	3 (2.05)
Overweight, N (%)	41 (28.08)
Class I obesity, N (%)	46 (31.50)
**Occupational activities**	
Domestic activities, N (%)	49 (33.56)
Gardening, Domestic activities, N (%)	69 (47.96)
Hobby, Domestic activities, N (%)	15 (10.27)
Dependent in performing ADL, N (%)	13 (8.90)

DD—demographic data

**Table 2:** Distribution according to etiology, presence of ventilatory defect, and level of dyspnea.

Etiology	N (%)
cardiac	43 (29.45)
pulmonary	90 (61.65)
other causes	13 (8.9)
**Ventilatory defect**	
OVD	52 (35.61)
RVD	26 (38.35)
MVD	12 (8.21)
Absence of ventilatory defect	56 (38.35)
**Modified BORG dyspnea scale**	
LEVEL 1	26 (17.80)
LEVEL 2	65 (44.52)
LEVEL 3	55 (37.67)

OVD—obstructive ventilatory defect, RVD—restrictive ventilatory defect, MVD—mixed ventilatory defect.

**Table 3:** Evaluation of functional limitation determined by dyspnea felt with the help of the LCADL test and walking distance (6MWT).

Parameters	Mean	SD	Min	Max	*p*-Value
LCADL 1 score	28.53	13.26	5.000	60.000	<0.001
LCADL 2 score	19.82	10.63	0.000	47.000	
6MWT 1 (m)	394.25	94.62	240.000	580.000	<0.001
6MWT 2 (at 3 months) (m)	470.83	106.27	250.000	678.000	

Min—Minimum; Max—Maximum, 6MWT 1—6 min walk test, first evaluation; 6MWT 2—6 min walk test, second evaluation; LCADL 1—London Chest Activity of Daily Living, first evaluation; LCADL 2—London Chest Activity of Daily Living, second evaluation; SD—std. deviation; m—meters.

**Table 4:** Statistics of the regression model.

Cases	Sum of Squares	df	Mean Square	F	*p*
6MWT (m)	428,132.743	1	428,132.743	614.781	*p* < 0.001
Residuals	100,977.757	145	696.398		

**Table 5:** Post hoc comparisons between baseline and final values of the 6MWT test.

initial Value	final Value	Mean Difference	SE	t	p_bonf_
6MWT1	6MWT2	76.582	3.638	24.795	<0.001

p_bonf_—*p* Bonferroni.

**Table 6:** Mean values obtained with the 6MWT test are presented differentially by etiology at baseline and 3-month reassessment.

Causes of Dyspnea	N	Initially − M ± SD	After 3 Months − M ± SD
cardiac	23	377.00 ± 90.38	449.00 ± 102.05
pulmonary	90	389.62 ± 99.286	468.55 ± 21.27
others	33	418.88 ± 81.443	492.24 ±93.13

M—mean; SD—standard deviation; N—number of patients

**Table 7:** Results of the post hoc analysis on the evolution of the 6MWT test according to etiology.

Causes of Dyspnea	Causes of Dyspnea	Mean Difference	SE	t	p_bonf 1_	p_bonf 2_
others	cardiac	42.561	26.779	1.589	0.343	0.407
others	pulmonary	26.472	20.063	1.319	0.567	0.824
cardiac	pulmonary	−16.089	23.035	−0.698	1.000	1.000

p_bonf1_—*p* Bonferroni initial; p_bonf2_—*p* Bonferroni final.

**Table 8:** Pearson correlation of LCADL score with 6MWT and age.

Variable	Coefficient	LCADL1	6MWT	Age
1. LCADL 1	*n*	—		
	Pearson’s r	—		
	*p*-value	—		
2. 6MWT	*n*	146	—	
	Pearson’s r	**−0.794**	—	
	*p*-value	<0.001	—	
3. Age	*n*	146	146	—
	Pearson’s r	**0.484**	**−0.615**	—
	*p*-value	<0.001	<0.001	—

6MWT—6 min walk test; LCADL1—London Chest Activity of Daily Living, first evaluation.

**Table 9:** Statistically significant predictors of walk test.

Model		Unstandardized	Standard Error	t	*p*
**H_1_**	Age	−1.818	0.913	−1.991	**0.049**
	LCADL 1	−3.818	0.421	−9.070	**<0.001**
	modified Borg scale	−36.680	9.815	−3.737	**<0.001**
	environment (u)	−1.560	9.826	−0.159	0.874
	marital status (single)	8.256	10.179	0.811	0.419
	occupational tasks (dependent on help)	−19.178	19.520	−0.982	0.328
	occupational tasks (gardening, domestic activities)	−12.285	12.764	−0.963	0.338
	occupational tasks (hobby, domestic activities)	−3.929	17.017	−0.231	0.818
	causes of dyspnea (cardiac)	33.231	14.549	2.284	**0.024**
	causes of dyspnea (pulmonary)	19.881	10.499	1.894	0.060

## Data Availability

The original contributions presented in this study are included in the article, further inquiries can be directed to the corresponding authors.
